# Melatonin and cortisol exhibit different circadian rhythm profiles during septic shock depending on timing of onset: a prospective observational study

**DOI:** 10.1186/s13613-018-0462-y

**Published:** 2018-12-04

**Authors:** Eleni N. Sertaridou, Ioanna G. Chouvarda, Konstantinos I. Arvanitidis, Eirini K. Filidou, George C. Kolios, Ioannis N. Pnevmatikos, Vasilios E. Papaioannou

**Affiliations:** 1 Intensive Care Unit, Alexandroupolis University Hospital, Democritus University of Thrace, 68100 Dragana, Alexandroupolis, Greece; 2 Laboratory of Computing, Medical Informatics and Biomedical Imaging Technologies, Faculty of Medicine, Aristotle University of Thessaloniki, Thessaloniki, Greece; 3 Laboratory of Pharmacology, Faculty of Medicine, Democritus University of Thrace, 68100 Dragana, Alexandroupolis, Greece

**Keywords:** Melatonin, 6-Sulfatoxymelatonin, Circadian rhythm, Cortisol, Septic shock, Critically ill patients

## Abstract

**Background:**

Septic shock has been found to disrupt circadian rhythms. Moreover, timing of onset has been associated with different circadian profiles in experimental studies.

**Results:**

In this prospective study, we enrolled 26 patients divided into two groups: Group A (*N* = 15) included subjects who had septic shock at the time of ICU admission and Group B (*N* = 11) included patients who developed septic shock during ICU admission. 6-Sulfatoxymelatonin (aMT6s) and cortisol levels were measured in urine samples every 4 h over a 24-h period. Two sets of samples were taken from Group A (entry/septic shock and exit) and three sets from Group B (entry, septic shock and exit). Mean, amplitude that is the difference between peak and mean values, as well as peak time, were estimated for both aMT6s and cortisol. In Group A, amplitude of aMT6s upon entry (septic shock) was reduced in relation to exit (437.2 ± 309.2 vs. 674.1 ± 657.6 ng/4 h, *p* < 0.05). Peak time occurred earlier (10:00 p.m. vs. 07:00 a.m, *p* < 0.05) and correlated with higher APACHE II score and longer ICU stay. In Group B, aMT6s mean values were significantly increased during septic shock (2492.2 ± 1709.1 ng/4 h) compared to both entry (895.4 ± 715.5 ng/4 h) and exit (1308.6 ± 1214.4 ng/4 h, *p* < 0.05 for all comparisons). Amplitude of aMT6s was also elevated during septic shock (794.8 ± 431.8 ng/4 h) in relation to entry (293.1 ± 275.9 ng/4 h, *p* < 0.05). Regarding cortisol rhythm in Group A, during septic shock amplitude was increased compared to exit (13.3 ± 31 ng/4 h vs. 8.7 ± 21.2 ng/4 h *p* < 0.05) and correlated with reduced hospital length of stay. In Group B, cortisol mean values and amplitude during septic shock (10 ± 5.3 and 3 ± 1.8 ng/4 h, respectively) were significantly reduced compared to both entry (30 ± 57.9 and 12.3 ± 27.3 ng/4 h) and exit (14.4 ± 20.7 and 6.6 ± 8.7 ng/4 h, *p* < 0.05 for all comparisons) and correlated with higher SOFA score and longer ICU and hospital stay.

**Conclusions:**

Septic shock induced inverse changes of aMT6s and cortisol circadian rhythm profiles both within and between different groups of patients, depending on timing of onset. Reduced rhythmicity was correlated with severity of disease and longer ICU stay.

## Background

Several biochemical, physiological and behavioral parameters exhibit periodical fluctuations. Circadian rhythms refer to self-sustained fluctuations with a period of approximately (*circa*) 1 day (*diem*) in various physiological functions. Different time keepers, meaning rhythmic signals from the environment (e.g., the change between day light and dark, meal times), can synchronize the endogenous rhythms (e.g., interchange between sleep and awakening). This 24-h period ‘circadian clock’ is developed to anticipate environmental changes associated with daylight cycles [1]. Serum melatonin, its urine metabolite 6-sulfatoxymelatonin (aMT6s), cortisol and core body temperature rhythms are considered as circadian biomarkers, controlled by a common circadian clock located in the hypothalamic Suprachiasmatic Nuclei (SCN) [2, 3].

Critically ill patients experience severe circadian deregulation associated with both systemic inflammation and Intensive Care Unit (ICU) environment [3]. Disruption of circadian rhythmicity can impair immune function, increasing incidence and severity of infection and worsening outcome [2, 4].

Many experimental studies suggest that loss of melatonin’s circadian rhythmicity negatively influences mortality in animals with septic [5, 6] or hemorrhagic shock [7]. Only a few clinical studies have reported that circadian rhythm disturbances are common among different groups of critically ill patients with [2, 3, 8–11] or without sepsis [4, 12]. However, most investigators have evaluated either serum melatonin fluctuations or urine aMT6s excretion in isolation. Rarely, authors studied combined urine aMT6s and cortisol excretion [8]. Furthermore, the comparisons were made between study and control groups, often consisted of healthy volunteers [11, 13].

In this study, we decided to examine the potential alterations of circadian rhythmicity of urine aMT6s and cortisol excretion in critically ill patients who were admitted in the ICU with septic shock or developed septic shock during their ICU stay, in a prospective fashion and on successive occasions (septic shock and recovery). Our primary purpose was to record different profiles of these biomarkers during different states of disease (septic shock versus recovery) in the same group of patients. Secondary outcomes of this study were considered the evaluation of potential correlations between the degree of circadian deregulation and sepsis severity, as well as different outcomes of interest, such as length of ICU and hospital stay and hospital mortality.

## Methods

This was a prospective observational study conducted at the General ICU of the University Hospital of Alexandroupolis, Greece. It was approved by the Hospital Ethics Committee, while written informed consent from patients or their next of kin was not necessary.

### Study design

Study subjects were recruited among critically ill patients admitted to the ICU between February 2015 and July 2017. The inclusion criteria were: (1) patients admitted with septic shock or developed septic shock during their ICU stay [14], (2) age > 18 and < 80 years, (3) ICU stay more than 48 h. Exclusion criteria were: (1) history of neurological or psychiatric disorders [15] or recent brain injury [16], (2) history of cancer [17], autoimmune disease or immunosuppressive therapy [18, 19], (3) alcohol or drug abuse [20, 21], (4) acute kidney injury [3, 11], (5) liver dysfunction [3, 11], (6) endocrine disorders or cortisol treatment [2] and (7) pregnant or postpartum women [15], since such cases are associated with sleep–wake cycle disruption and circadian deregulation.

Patients were allocated into two groups: Patients who had septic shock at the time of ICU admission consisted Group A. Group B included critically ill patients (trauma and major abdominal surgery), who developed septic shock during ICU admission. Patients from Group A during septic shock and from Group B during both the entry phase and septic shock were deeply sedated with propofol and remifentanil, mechanically ventilated and treated with vasoactive drugs when indicated [14]. None from these patients received hydrocortisone during the whole study period. Patients during sedation had their eyes closed throughout the whole 24-h sampling period. To avoid artifacts by artificial light during night, lights were turned off during the night hours except during the nursing rounds. The ICU environment allowed regular changes between night and daylight for awaked subjects [11]. All patients were followed until discharge from the hospital, to assess ICU and in-hospital length of stay and mortality.

### Data collection and measures

Demographic, clinical and biochemical data were collected from the patient’s electronic medical record. Severity of disease upon admission was assessed using both the Acute Physiology and Chronic Health Evaluation (APACHE II) score and Simplified Acute Physiology Score II (SAPS II). Furthermore, daily severity of illness was evaluated with the Specific Organ Failure Assessment score (SOFA) [14].

#### Circadian rhythm of aMT6s and cortisol excretion

We collected 24-h urine samples from all patients within less than 24 h of admission, onset of septic shock and before discharge. Two milliliters of urine was collected through an indwelling bladder catheter every 4 h over a 24-h period beginning from the time of enrollment. Thus, 6 urine samples were obtained from every patient over each 24-h period of sampling. For patients who had septic shock at the time of ICU admission, sampling occurred at enrollment during septic shock (entry phase) and within 24 h before discharge from the ICU (recovery/exit phase). For those who developed septic shock during ICU admission, sampling occurred at admission (entry phase), at the occurrence of septic shock (sepsis phase) and within 24 h before discharge from the ICU (recovery/exit phase). The total urine volume of the 4-h period was also recorded, and the samples were immediately frozen at − 80 °C until assayed. Concentrations of aMT6s were measured by competitive Enzyme-Linked Immune Sorbent Assay (ELISA) according to the manufacturer’s instructions (Melatonin-sulfate Urine ELISA kit, IBL International GmbH, Germany). We determined urinary aMT6s level, as an accepted noninvasive method to study melatonin circadian rhythmicity, since it has been shown to be highly reflective of serum melatonin concentration [2]. Urine concentration of cortisol was measured by Fluorescence Polarization Immunoassay (FPIA) (AXSYM system, Abbott). Urinary excretion of both aMT6s and cortisol was estimated from the calculated product of their concentration/ml by urine volume for each 4-h interval over a period of 24 h. Mean, amplitude that is the difference between peak and mean values and peak time that reflects the time of peak value in relation to midnight were considered markers of circadian rhythmicity [18].

#### Statistical analysis

There were two main arms in this analysis of circadian parameters: (1) to compare individual mean, amplitude and peak time values [22] observed during septic shock to those obtained before discharge in patients who had septic shock at the time of ICU admission and similarly, values upon ICU entry, septic shock development and before discharge in subjects who developed septic shock during ICU admission and (2) to evaluate which parameters correlate with SOFA score during septic shock, severity of disease upon admission, as well as different clinical outcomes of interest, such as hospital mortality, length of ICU and hospital stay.

We applied the Lilliefors test as an adaptation of Kolmogorov–Smirnov test for assessing the null hypothesis that data come from a normally distributed population. None from the studied circadian variables was found to follow a normal distribution. In this respect, nonparametric statistical analysis via Wilcoxon test (2 classes) and Kruskall–Wallis test (3 classes) was used. The independent Student’s *t * - test was used to compare the demographic characteristics between both groups. The package corrplot was employed for an enhanced visualization of correlation analysis among the circadian parameters and clinical variables [23]. Due to multiple comparisons (12) and in order to protect from type I error (false positive results), a post hoc Bonferroni correction was conducted, getting an adjusted *p* value after dividing the original *p* value (0.05) by the number of analyses performed. In our case, since we evaluated 6 circadian (mean, amplitude, peak time for both aMT6s and cortisol) and 6 clinical parameters, the new corrected *p* value was 0.004 (0.05/12). All analyses were performed with R version 3.4.4.

## Results

**Fig. 1:**
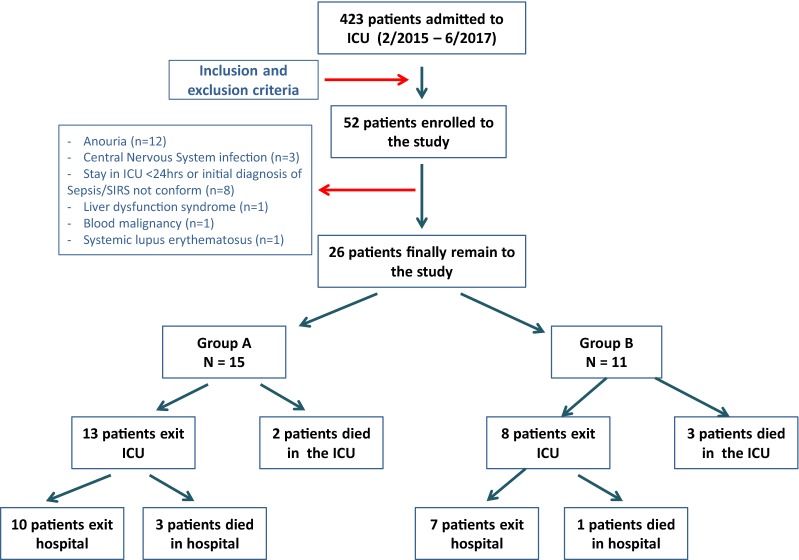
Flowchart. Flowchart of study design and patients’ characteristics who were enrolled in the study

During the study period, 423 patients in total were admitted to the ICU. Fifty-two fulfilled criteria for enrollment. Twenty-six patients were finally excluded (Fig. 1). The characteristics of the patients who were included in the analysis at the time of enrollment are listed in Table 1. Two patients from Group A and 3 from Group B died during ICU hospitalization; however, urine samples were collected and analyzed 1–2 days before death. APACHE II, SAPS II, SOFA scores, as well as length of hospital and ICU stay, did not differ between and within the 2 groups.

**Table 1:** Patients’ characteristics

Parameter	Group A (*N *= 15)	Group Β (*N *= 11)	*p* value
Age (years)	60.4 ± 25.3	60.9 ± 20.1	0.823
Sex, male/female	9/6	8/3	0.877
Diagnosis	Pneumonia (*n* = 7)	Multi-trauma patients (*n* = 5)	
	Abscess (*n* = 1)	Aortic aneurysm rapture (*n* = 2)	
	Intra-abdominal sepsis (*n* = 2)	Acute pulmonary edema (*n* = 2)	
	Bacteraemia (*n* = 4)	Postoperative respiratory failure (*n* = 1)	
	Urinary tract sepsis (*n* = 1)	Postsurgical stabilization (*n* = 1)	
APACHE II score	20.9 ± 8.2	18.9 ± 4	0.653
SAPS II score	44.4 ± 16.6	43.1 ± 15.1	0.642
Sepsis SOFA score	7.3 ± 3.3	7.9 ± 3.4	0.454
Ramsay score	4.7 ± 2.5	5.5 ± 1.8	0.547
Time of septic shock onset	Upon admission (12.8 ± 8.7 h)	7.5 ± 3.4 days	
Length of ICU stay, (days)	10.1 ± 10.2	18.5 ± 11.8	0.234
Died in ICU	2	3	
Cause of death in ICU	Septic shock—MODS (*n* = 2)	Multi-relapsed septic episodes—MODS (*n* = 2)	
		AKI—liver dysfunction (*n* = 1)	
Length of hospital stay, (days)	26 ± 21.1	26.4 ± 12.2	0.742
Died in hospital	3	1	
Cause of death in hospital	Septic shock—MODS (*n* = 1)	Intra-abdominal sepsis (*n* = 1)	
	Intra-abdominal sepsis—(*n* = 1)		
	Hemorrhagic shock (*n* = 1)		

Values are (Mean ± SD), *p* value for statistical comparison by t-test

*p* values < 0.05 were considered statistically significant

*APACHE II* Acute Physiology and Chronic Health Evaluation, *h* hours, *ICU* Intensive Care Unit, *MODS* Multi-Organ Dysfunction Syndrome, *NS* no significant, *SAPS II* Simplified Acute Physiology Score, *SOFA* Specific Organ Failure Assessment

### Urinary excretion of aMT6s

**Fig. 2:**
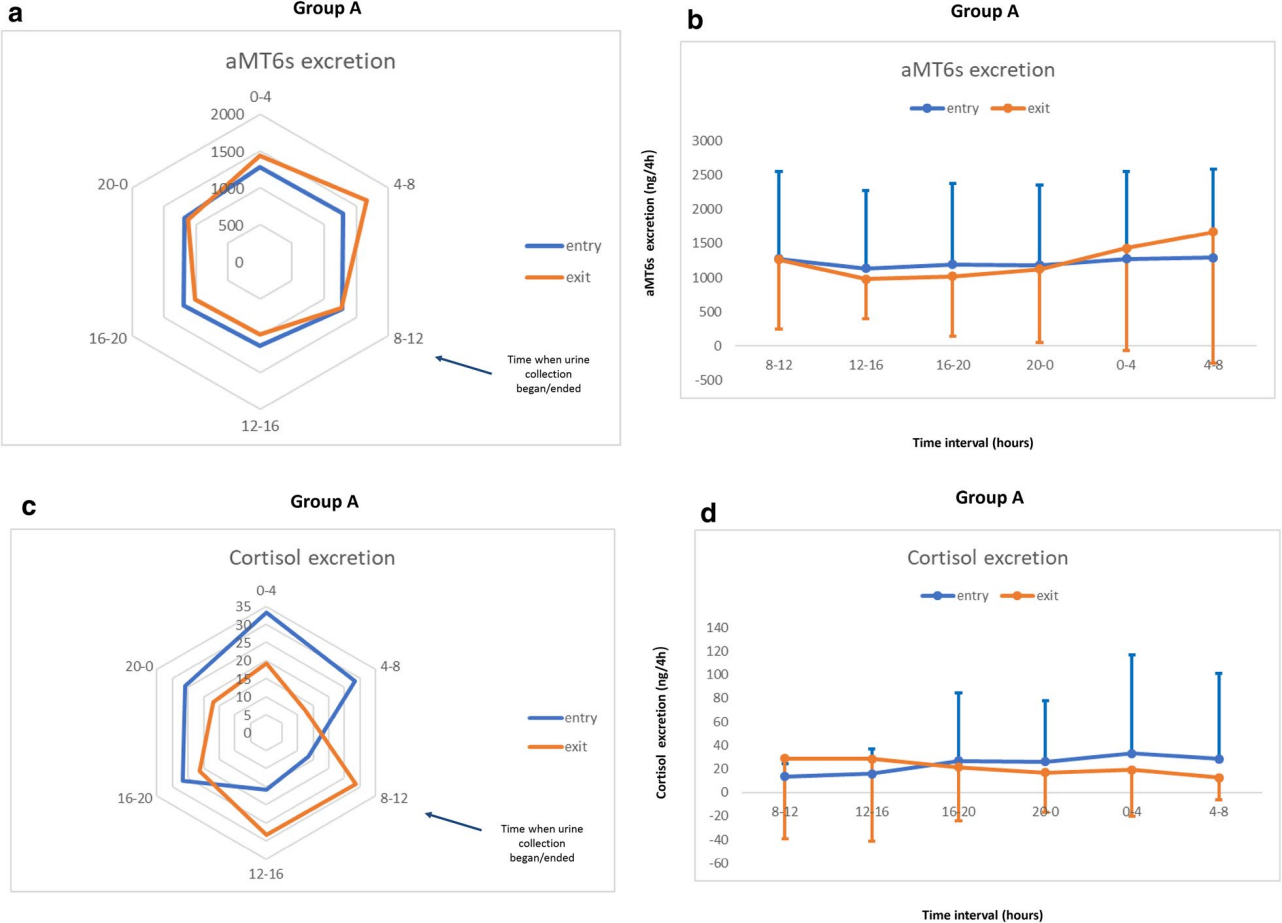
Radar plots and longitudinal trends of aMT6s and cortisol urine excretion in patients who had septic shock at the time of ICU admission (Group A). **a** Radar plots of urinary aMT6s excretion mean values (ng) per 4-h intervals upon entry and exit and within a 24-h period in patients who had septic shock at the time of ICU admission (*N* = 15). The value 0 corresponds to midnight. The blue arrow indicates when urine collection began/ended for all patients. aMT6s = 6-sulfatoxymelatonin. **b** Longitudinal 24-h profiles of urinary melatonin excretion (aMT6s) mean values per 4-h intervals upon entry and exit in patients who had septic shock at the time of ICU admission. Error bars represent standard deviations (SDs). The value 0 on x axis corresponds to midnight. **c** Radar plots of urinary cortisol excretion mean values (ng) per 4-h intervals upon entry and exit and within a 24-h period in patients who had septic shock at the time of ICU admission. The value 0 corresponds to midnight. The blue arrow indicates when urine collection began/ended for all patients. **d** Longitudinal 24-h profiles of urinary cortisol excretion mean values per 4-h intervals upon entry and exit in patients who had septic shock at the time of ICU admission. Error bars represent standard deviations (SDs). The value 0 on x axis corresponds to midnight

**Fig. 3:**
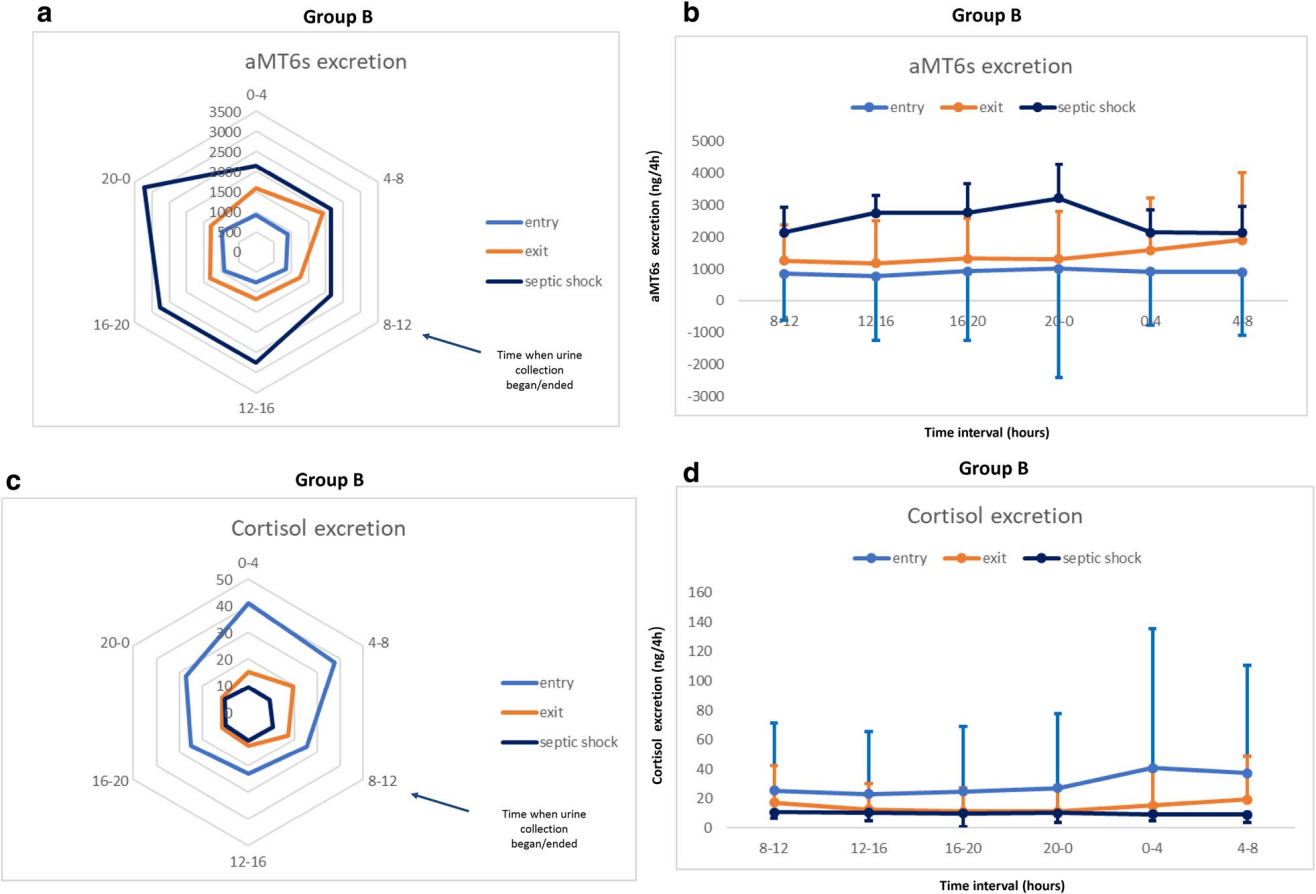
Radar plots and longitudinal trends of aMT6s and cortisol urine excretion in patients who developed septic shock during ICU admission (Group B). **a** Radar plots of urinary aMT6s excretion mean values (ng) per 4-h intervals upon entry, septic shock and exit and within a 24-h period in patients who developed septic shock during ICU admission (*N* = 11). The value 0 corresponds to midnight. The blue arrow indicates when urine collection began/ended for all patients. aMT6s = 6-sulfatoxymelatonin. **b** Longitudinal 24-h profiles of urinary aMT6s excretion mean values per 4-h intervals upon entry, septic shock and exit in patients who developed septic shock during ICU admission. Error bars represent standard deviations (SDs). The value 0 on x axis corresponds to midnight. aMT6s = 6-sulfatoxymelatonin. **c** Radar plots of urinary cortisol excretion mean values per 4-h intervals upon entry, septic shock and exit and within a 24-h period in patients who developed septic shock during ICU admission. The value 0 corresponds to midnight. The blue arrow indicates when urine collection began/ended for all patients. **d** Longitudinal 24-h profiles of urinary cortisol excretion mean values per 4-h intervals upon entry, septic shock and exit in patients who developed septic shock during ICU admission. Error bars represent standard deviations (SDs). The value 0 on x axis corresponds to midnight

The 24-h profile of aMT6s urine excretion in patients who had septic shock at the time of ICU admission is shown in Fig. 2a and b. Thus, during septic shock amplitude was lower and the peak time occurred earlier in relation to recovery (Table 2). In patients who developed septic shock during ICU admission, upon ICU entry there was an abrogation of aMT6s circadian rhythm. During septic shock, all aMT6s circadian variables were significantly increased, whereas peak values occurred significantly earlier compared with both entry and exit (Table 2, Fig. 3a, b). The rhythm tended to normalize upon discharge, but remained abnormal with a peak value shifted toward early morning hours.

**Table 2:** Urinary aMT6s and cortisol excretion

	Group A		Group B		
	Entry (septic shock) mean (SD)	Exit (recovery phase) mean (SD)	Entry mean (SD)	Septic shock mean (SD)	Exit (recovery phase) mean (SD)
aMT6s mean (ng/4 h)	1204.9 (873.7)	1264.4 (983.8)	895.4 (715.5)	2492.2*^$^ (1709.1)	1308.6 (1214.4)
aMT6s ampl (ng/4 h)	437.2* (309.2)	675.1 (657.6)	293.1 (275.9)	794.8*^$^ (431.8)	954.5 (943.1)
aMT6s peak time (h)	11:00* p.m.	09:00 a.m.	06:00 a.m.	10:00*^$^ p.m.	07:00 a.m.
Cortisol mean (ng/4 h)	23.8 (48.7)	20.7 (43.6)	30 (57.9)	10*^$^ (5.3)	14.4 (20.7)
Cortisol ampl (ng/4 h)	13.3* (31)	8.7 (21.2)	12.3 (27.3)	3*^$^ (1.8)	6.6 (8.7)
Cortisol peak time (h)	01:00* a.m.	10:00 a.m.	01:00 a.m.	06:00*^$^ p.m.	06:00 a.m.

*ampl* amplitude (difference between peak and mean values)

* Statistically significant (*p* < 0.05) for comparisons between septic shock and exit

^$^Statistically significant (*p* < 0.05) for comparisons between septic shock and entry

During septic shock in Group A (Table 3), higher aMT6s mean values were correlated with lower hospital mortality (*r* = − 0.5, *p* < 0.004). Furthermore, patients with higher APACHE II score upon admission had earlier peak urine aMT6s excretion (*r* = 0.6, *p* < 0.004), which was subsequently correlated with longer ICU stay (*r* = 0.6, *p* < 0.004). In addition, patients with high SOFA score during septic shock exhibited lower aMT6s mean (*r* = − 0.7, *p* < 0.004) and amplitude (*r* = − 0.8, *p* < 0.004) values during recovery (Table 3). In Group B, high APACHE II and SAPS II scores in entry were associated with a significant shift of peak time of aMT6s during septic shock (*r* = 0.9, *r* = 0.9, *p* < 0.004, respectively), which was subsequently correlated with longer ICU and hospital stay (*r* = 0.6, 0.6, *p* < 0.05, respectively), as well as increased hospital mortality (*r* = 0.7, *p* < 0.004) (Table 4).

**Table 3:** Significant correlations in Group A between circadian variables, severity of illness scores and different outcomes of interest (ICU & hospital LOS and hospital mortality)

	Entry (septic shock)						Exit (recovery phase)					
	APACHE II	SOFA	SAPS II	ICU LOS	Hospital LOS	Hospital mortality	APACHE II	SOFA	SAPS II	ICU LOS	Hospital LOS	Hospital mortality
aMT6s mean						− 0.5*		− 0.7*				
aMT6s ampl								− 0.8*				
aMT6s peak time	0.6*			0.6*					0.5			
Cortisol mean					− 0.5	− 0.6*		− 0.5				− 0.5
Cortisol ampl						− 0.5*						− 0.6
Cortisol peak time				0.5	0.6			0.5				− 0.5

All correlations were found statistically significant with a *p* value < 0.05

*ampl* amplitude (difference between peak and mean values), *LOS* length of stay

* Correlations who passed the Bonferroni correction test with a *p* value < 0.004 (*p* corrected = 0.05/12)

**Table 4:** Significant correlations in Group B between circadian variables, severity of illness scores and different outcomes of interest (ICU & hospital LOS and hospital mortality)

	Entry						Septic shock						Exit (recovery phase)					
	APACHE II	SOFA	SAPS II	ICU LOS	Hospital LOS	Hospital mortality	APACHE II	SOFA	SAPS II	ICU LOS	Hospital LOS	Hospital mortality	APACHE II	SOFA	SAPS II	ICU LOS	Hospital LOS	Hospital mortality
aMT6s mean								0.6		0.5	0.5							
aMT6s ampl							–	0.5	–									
aMT6s peak time							0.9*		0.9*	0.6	0.6	0.7*						
Cortisolmean	0.5							− 0.8	0.5	− 0.9*	− 0.8*	0.6*			− 0.5			
Cortisol ampl	0.5			0.5	0.5			− 0.6		− 0.6*	− 0.7*	0.6*			− 0.6			
Cortisol peak time				0.5				− 0.95		− 0.9*	− 0.7*	0.6*	0.5	0.6				

All correlations were found statistically significant with a *p* value < 0.05

*ampl* amplitude (difference between peak and mean values), *LOS* length of stay

* Correlations who passed the Bonferroni correction test with a *p* value < 0.004 (*p* corrected = 0.05/12)

### Urinary excretion of cortisol

The 24-h profile of cortisol urine excretion in patients who had septic shock at the time of ICU admission is shown in Fig. 2c and d. It seems that amplitude was significantly increased during septic shock with a significant shift of peak time toward late night hours in relation to the recovery phase (Table 2). In patients who developed septic shock during ICU admission (Table 2, Fig. 3c, d), there was a significant reduction in cortisol mean and amplitude values during septic shock compared to both entry and exit. The rhythm tended to normalize upon discharge. In Group A, during septic shock (Table 3) patients with higher cortisol mean and amplitude had reduced hospital mortality (*r* = − 0.6, *r* = − 0.5, *p* < 0.004, respectively). Moreover, earlier cortisol peak time correlated with longer ICU and hospital stay (*r* = 0.5, *r* = 0.6, *p* < 0.05, respectively). In Group B, reduced mean and amplitude of cortisol rhythm, as well as peak time delay toward afternoon hours during septic shock, were significantly correlated with longer ICU and hospital stay and increased hospital mortality (*p* < 0.004, Table 4). Finally, no significant correlations were found between duration of ICU stay prior to development of septic shock and different circadian alterations observed.

## Discussion

Circadian rhythms in critically ill patients are significantly disturbed due to environmental factors (lack of change between light and dark, noise), sleep deprivation, different medications or disease itself. Such circadian deregulation usually appears with a change in amplitude of the 24 h cycle of different circadian biomarkers, such as melatonin and cortisol urine excretion, characterized by an almost flattened time series with reduced fluctuations. Moreover, peak time can display a phase shift and occur at different than the usual time of day or night [24]. It has been postulated that ‘loss of amplitude might impair the capacity for adaptation,’ whereas phase shift ‘uncouples maximum function from peak demand’ [25].

In this study, we investigated for the first time concurrently the alterations of circadian rhythmicity of both aMT6s and cortisol urine excretion in critically ill patients during septic shock with different timing of onset. Although the time of an event (time of day) is different from individuals’ ‘circadian time,’ we decided to evaluate circadian fluctuations in patients developing septic shock upon and during ICU admission in order to assess potential differences between acute and acute-on-chronic inflammatory states. However, we enrolled patients during morning hours for practical reasons and not at the exact time of septic shock onset. Experimental studies have suggested that during acute inflammation, high levels of pro-inflammatory cytokines (IL-6, tumor necrosis factor alpha—TNFα) diminish amplitude and delay peak values of aMT6s through decreased clock gene expression [26]. On the other hand, prolonged inflammatory response during critical illness may induce an immune-suppressed state where melatonin and cortisol rhythm profiles might exhibit different patterns of change during an adverse event, such as development of septic shock [27].

The main findings of our study were that in patients who had septic shock at the time of ICU admission, aMT6s and cortisol circadian rhythms were altered in an inverse way. Thus, although mean values did not change significantly, amplitudes of aMT6s were reduced, whereas peak time occurred earlier in relation to recovery. Conversely, cortisol amplitudes were increased with a shift in peak time toward late night hours. A correlation between the severity of illness and the degree of aMT6s circadian rhythm disruption was noted, whereas patients with high SOFA score during septic shock exhibited lower mean and amplitude values upon exit. Regarding patients who developed septic shock during ICU admission, both aMT6s and cortisol exhibited inverse patterns of change, since all aMT6s circadian variables were significantly increased, whereas those of cortisol were significantly reduced.

Melatonin is synthesized by the pineal gland upon sympathetic stimulation of pinealocytes. Thus, darkness stimulates retinal photoreceptors to release norepinephrine, which subsequently through both *α*1 and *β*1 adrenergic receptors stimulate synthesis of melatonin. Pinealocytes convert tryptophan to melatonin via the intermediates 5-hydroxytryptophan and serotonin. Serotonin is then converted to N-acetylserotonin by N-acetyl-transferase (NAT) and finally to melatonin. NAT is the rate limiting step in the synthesis of melatonin and exhibits increased nocturnal activity [28]. Melatonin is increased during sleepiness and decreased during wakefulness and thus conveys the information of nighttime to the organism. In healthy humans, melatonin begins to rise at 08:00 pm, peaks at about 2.00 am and then returns to normal by 7.00 am. It also plays the role of an endogenous synchronizer, able to stabilize circadian rhythms and maintain their mutual phase relationships [28].

Both endogenous melatonin and exogenous melatonin are metabolized by the liver to 6-hydroxymelatonin with subsequent conjugation to 6-sulfatoxymelatonin with sulfuric acid. Conjugated metabolites are renally excreted, and aMT6s parallels melatonin secretion and therefore reflects circulating melatonin levels. Orally administered melatonin exhibits wide variability of its bioavailability due to inter-individual differences in the activity of the cytochrome P450 1A2 (CYP1A2), which is responsible for its hepatic metabolism. Its elimination half-life (T1/2) is approximately 30-45 min, time to maximal plasma/serum concentration (Tmax) is around 50 min, and its bioavailability is reported to be approximately 15% [28, 29].

Target cells express specific high affinity receptors, namely, MT1/MT2 on plasma membrane that trigger intracellular signaling by adenylate cyclase or G proteins [28]. Melatonin in general is considered an effective anti-inflammatory agent due to inhibition of nitric oxide synthase, with consequent reduction in peroxynitrite formation, stimulation of different antioxidant enzymes and suppression of TNF-α production [28, 30]. In addition, melatonin has also an extra-pineal source since different gastrointestinal and immune competent cells synthesize melatonin, which has a peripheral activity, such as protection against reperfusion injury in gut mucosa [31]. Melatonin produced by these organs is not regulated by circadian cycles, but corresponds to different signals, such as inflammatory stimuli.

In this respect, different authors have found that pineal melatonin is effective within low levels (nM–pM range) [32, 33]. Such ‘chronobiotic’ levels inhibit both rolling and adherence of leukocytes to the endothelial layer and thus decrease capillary leak and unnecessary inflammatory response. On the contrary, extra-pineal melatonin produced by local immune competent cells acts in a paracrine or autocrine manner as anti-inflammatory mediator in much higher concentrations (mM range) [32, 34]. In addition, numerous studies have confirmed that melatonin is rather an immunomodulator molecule [1, 35], behaving as either an immunostimulant under basal or immunosuppressed conditions, or displaying anti-inflammatory properties during transient or chronic exacerbated immune responses. Consequently, it has been described as an ‘immunologic buffer’ due to its pleiotropic effects on immune function [35].

Corticosteroids with their anti-inflammatory actions also enhance melatonin pineal production. Thus, in chronic inflammatory disorders, adrenal gland-derived glucocorticoides restore the nocturnal rise of melatonin [27]. Nevertheless, during acute stress, corticosteroids might decrease the activity of N-acetyl-transferase, and hence, raised levels of cortisol may negatively influence synthesis of melatonin [36]. In addition, peak plasma melatonin levels occur when cortisol levels are at their lowest.

In our study, since patients in the two groups did not differ in terms of APACHE II, SAPS II and SOFA score during septic shock, circadian deregulation might be attributed to different effects of the time onset of shock states on immune response. In this respect, circadian profiles of aMT6s and cortisol in patients who had septic shock at the time of ICU admission seem to reinforce previous findings regarding the potential impact of acute inflammation on loss of circadian rhythmicity [11]. Thus, septic shock was associated with a nonsignificant decrease in aMT6s mean values and a significant decrease in its amplitude compared to exit. At the same time, cortisol profile exhibited inverse changes. Pontes et al. [37] have shown in human females with and without mastitis that pro-inflammatory cytokines block pineal endocrine production of melatonin and switch it to paracrine production from peripheral immunocytes present in inflamed tissues, defining the immune-pineal axis. In this respect, our results could be due either to the reduced pineal production of melatonin during acute stress or to an activation of the hypothalamic–pituitary–adrenal (HPA) axis. Moreover, it has been recently shown that during acute inflammation the simultaneous activation of both glucocorticoid and adrenergic receptors may reduce the synthesis of melatonin during night [38].

Regarding patients who developed septic shock around the 7th day of ICU stay, inverse changes were found in circadian markers with significant increase in aMT6s and parallel decrease in cortisol’s mean and amplitude values compared to both entry and exit. Since Group B included mainly postsurgical patients, the initial postoperative reduction in melatonin secretion and reduced rhythmicity that was observed in our patients at the time of admission to the ICU replicates similar findings from other studies [39]. It has been proposed that postoperative stress might be associated with a potential increase in melatonin’s consumption as its antioxidant properties could be utilized for neutralizing the reactive oxygen species induced by surgery [28]. Moreover, raised cortisol levels might negatively influence synthesis of melatonin. During development of septic shock and since critically ill postsurgical patients seem to remain in an immunosuppressive TH2 state [28], an increased pineal production of melatonin could be considered a physiological counteracting response due to its immunostimulant properties [35]. Thus, significant increase in circadian measures of aMT6s during septic shock might reflect either an adaptive response or the effects of suppressed HPA axis in the context of a potential adrenal insufficiency [38], associated with an abrogation of cortisol’s rhythmicity that was found in our study. During recovery, all circadian markers seem to return to normal.

Our findings are in line with those of Verceles et al. [3], who reported a disruption of circadian rhythms of aMT6s in septic critically ill patients, whereas Perras found an inverse correlation between single nocturnal melatonin concentration at 2.00 a.m and APACHE II score, in 14 patients with severe sepsis during their first night in ICU [13]. However, our results regarding Group B seem to contradict findings from the study of Mundigler et al. [11], who found slightly increased mean values and significantly decreased amplitude of aMT6s with a peak time delay in patients who developed severe sepsis approximately 11 days in average after ICU stay and before study entry, compared to nonseptic critically ill subjects and controls. The reasons could be that study populations were different since most of the septic patients from Mundigler study were admitted to the ICU due to medical causes, whereas in our case, Group B included mostly postsurgical patients. In addition, comparisons between patients with different levels of stress or time of ICU stay before septic shock onset cannot capture intra-patient circadian rhythm dynamics affected by sepsis that was the main objective of our study.

Plenty of factors pertinent to the ICU environment and the management of critically ill patients have been demonstrated to affect the rate of melatonin secretion. Specifically, the continuous exposure of ICU patients to artificial light, constant noise and the frequent medical interventions are associated with an impairment of melatonin’s circadian rhythm [40]. Nevertheless, Perras and coworkers [41] showed that exposure to 10.000 lx of light for 1 h failed to influence circadian rhythms in a population of septic critically ill patients. Similarly, Verceles et al. [3] found that light with a maximum intensity of 650 lx despite exhibiting diurnal variations was incapable to entrain aMT6s circadian rhythm, in a cohort of sedated critically ill septic patients. According to the authors, sepsis per se could alter circadian output and diminish sensitivity to light entrainment, since melatonin over-production might be a physiological response to critical illness.

Medications constitute a potential confounder in circadian rhythm analysis of critically ill patients. In this respect, opioids show significant interspecies differences [2], whereas benzodiazepines have been found to decrease nocturnal and increase day time melatonin excretion in healthy adults [42]. In our study, we used remifentanil, which has not been found to affect melatonin’s secretion in mechanically ventilated patients [43]. Regarding sedation with propofol, there are only a few experimental data that have shown a potential peak time delay of melatonin after general anesthesia [44].

Concerning adrenergic agents, although catecholamines could have affected circadian patterns of change in our patients, it has been postulated by Parfitt and Klein [45] that the sympathetic pathway of reuptake of norepinephrine protects against the inappropriate increase in pineal melatonin production during stress, something that might reflect an adaptive response of the immune-pineal axis. In addition, human pineal gland seems to display poor responsiveness to circulating catecholamines [46]. Different studies in patients suffering from congestive heart failure have demonstrated that prolonged exposure to plasma catecholamines might decrease susceptibility to noradrenaline through down-regulation of beta-adrenergic receptors not only in myocardium, but also in the pineal gland [47]. In this respect, lipophilic beta blockers that have been found to inhibit pineal release of melatonin in healthy subjects [48] do not further suppress melatonin production in coronary artery disease patients. Since there are no data in septic critically ill patients so far, we suggest that the increased stress of our patients and a potential down-regulation of adrenergic receptors that has been observed during critical illness might limit counteracting effects of both vasopressors and beta blockers on melatonin release. Moreover, and even though beta blockers were not used during timing of measurements, all patients in our study received esmolol, a nonlipophilic agent, before and after extubation, as well as before performance of tracheotomy.

Mechanical ventilation has been associated with sleep deprivation and circadian deregulation, mainly due to patient-ventilator asynchrony, as well as hyper-assistance resulting in central apneas during night [8, 9, 49]. Parthasarathy and Tobin described significantly greater sleep fragmentation during pressure support ventilation (PSV) compared with assist-control ventilation (ACV) in sedated critically ill patients [49]. Olofsson and colleagues [8] studied melatonin levels in both blood and urine, for 3 consecutive days in 8 critically ill patients under sedation and mechanical ventilation. They were able to show that the circadian rhythm of melatonin release was abolished in all but one patient, whereas no correlation was found between melatonin levels and level of sedation, estimated with bispectral index (BIS). Frisk and colleagues [9] studied melatonin and cortisol as circadian biomarkers of 16 sedated and under ventilator support patients, during their whole ICU hospitalization. They found a statistically significant hyposecretion of aMT6s during mechanical ventilation, a significant increase upon adrenergic stimulation, overall high cortisol excretion and finally a disturbed diurnal rhythm of both these hormones in 75% of all patients. However, only two 12-h urine samples were collected and compared between the daytime and nighttime, reducing the chance of detecting daily fluctuations.

Gehlbach and coworkers showed that in a cohort of both septic and nonseptic sedated patients under ventilatory support, sleep/wake rhythm was disturbed, whereas normal sleep/wake cycles lacked a daily variation throughout the whole ICU stay [10]. In addition, most of the patients had preserved, but phase delayed excretion of aMT6s with reduced amplitude. According to the authors, episodic and sporadic ‘sleep-like states’ over a 24-h cycle could be responsible for the reduced amplitude and peak time delay of urine aMT6s. Nevertheless, urine collection was made only upon enrollment and concluded 24 or 48 h later. Moreover, in Mundlinger’s study, nonseptic and sedated ICU patients experienced a normal circadian profile of melatonin excretion, implying that immune response might be the most important stressor of circadian clock [11]. Furthermore, Danilenko and colleagues suggested that sleep per se remains a weak time keeper in humans without a concomitant change in the light/dark cycle [50].

Finally, in the study of Dessap et al. [51], delirium at the initiation of weaning from mechanical ventilation was associated with an alteration in the circadian rhythm of melatonin excretion during the first 24 h and a reduced probability of successful extubation.

The conflicting results of these studies are best explained by methodological differences, such as severity of disease, presence of sepsis, intensity of light exposure, level of sedation, vasopressor administration, modes of mechanical ventilation or different frequencies of urine collection for assessing excretion of aMT6s.

Our study was not designed to investigate the potential impact of different medications or therapeutic interventions on circadian rhythmicity. However, since many external time keepers that normally entrain central circadian clocks are absent or severely disturbed in the ICU setting, we cannot rule out their impact on circadian deregulation that was found in our patients. In any case, we hypothesized that in accordance with the findings of Verceles’ study [3], septic and sedated patients exhibit diminished sensitivity to light entrainment, whereas circadian alterations might reflect individual immune–circadian interactions. In this respect, and to avoid artifacts by altered light cycle changes, we implemented the protocol of Mundigler and colleagues [11]. In addition, both groups were under ACV during night, whereas we performed early tracheotomy in most of our patients and implemented a daily sedation interruption protocol.

A major limitation of our study is the lack of a reliable control group since every patient was considered as control of himself, whereas comparisons were made within patients upon admission, during ICU stay and before discharge. In this case, lack of different stressors during recovery, such as mechanical ventilation, or medications affecting circadian rhythmicity, might influence accuracy of our findings. However, most of the studies published so far aimed at cross-sectional assessments of circadian rhythm profiles between different groups of patients and have already suggested a negative impact of sepsis on circadian clocks. Nevertheless, comparisons between subjects with different levels of stress could be misleading since they fail to capture individuals’ ‘circadian time’ dynamics and its evolution over time. Furthermore, in the study of Mundigler and colleagues [11], circadian rhythm assessment of septic patients during recovery exhibited a tendency toward a slightly restored circadian pattern in some individuals, but lack of significant rhythm in any patient, indicating that disease itself and not ICU environment might be responsible for such findings. Similarly, in the present study a high SOFA score during septic shock was associated with reduced rhythmicity of aMT6s before discharge in patients who had septic shock at the time of ICU admission.

Duration of ICU hospitalization before development of septic shock could also influence circadian rhythmicity due to the negative impact of sedation and mechanical ventilation on urine excretion of aMT6s, as has been suggested from other studies with different design and patients’ characteristics [8–10]. However, a significant increase in mean and amplitude values of aMT6s during septic shock compared to both admission and recovery was found in our study. Thus, we suppose that our results might reflect different patterns of change of the immune-pineal-adrenal axis during stress, rather than any other prior therapeutic intervention. In addition, duration of illness prior to developing septic shock was not significantly correlated with any circadian alteration observed.

Other minor limitations of our study include small sample size and assessment of urinary aMT6s and cortisol excretion only during 24 h. Moreover, multiple correlations performed (Tables 3, 4) might suffer from increased error type I (false positives), but many of them remained significant even after applying a Bonferroni correction. In addition, we consider our data set is comparable with most of the similar studies evaluating circadian deregulation in septic ICU patients. However, we cannot confirm if different circadian profiles represent an adaptive or maladaptive counteracting mechanism during septic shock.

Finally, we suggest that since patients did not differ in terms of severity of illness, correlations found between circadian deregulation and different clinical outcomes might reflect a potentially negative impact of septic shock on biologic clocks with subsequent loss of ‘permissive homeostasis’ [1, 24]. In this respect and as has been suggested, ‘lack of appropriate anticipation of periods with increased stress or energy demands might negatively affect the function of individual cells, organ systems or the whole organism’ [24].

## Conclusions

Despite that association does not mean causality, the present study revealed significant alterations in both aMT6s and cortisol circadian rhythm during development of septic shock. However, we cannot rule out potential effects of ICU environment, sedation and vasoactive medications on circadian alterations observed. In addition, increased intra-individual and inter-individual variability limits generalization of our results to different groups of patients, whereas further investigations are required to investigate the pathophysiological and potential clinical implications of our findings.

## Abbreviations

Ampl: amplitude
aMT6s: 6-sulfatoxymelatonin
APACHE II: Acute Physiology and Chronic Health Evaluation II
ELISA: Enzyme-Linked Immune Sorbent Assay
FPIA: Fluorescence Polarization Immunoassay
HPA: hypothalamic–pituitary axis
ICU: Intensive Care Unit
MODS: Multi-Organ Dysfunction Syndrome
NAT: N-acetyl-transferase
SAPS II: Simplified Acute Physiology Score II
SCN: Suprachiasmatic Nuclei
SOFA: Specific Organ Failure Assessment
